# Atlanta Academy of Medicine

**Published:** 1877-01

**Authors:** J. S. Todd

**Affiliations:** Reporter


### Reports of Societies.

### ATLANTA ACADEMY OF MEDICINE.

J. S. TODD, M.D., Reporter.

Atlanta, Nov. 15, 1876.

Academy met, Dr. J. G. Westmoreland presiding.

Dr. J. P. Logan reported a case of embolic hemiplegia from aortic aneurism, which has caused a complete paralysis of the right side of the body, the tongue included. His power over the latter organ was, however, being restored, as he could now project it slightly. He first saw the case some four or five years since. He then complained of some difficulty of breathing, and there was slight cough and other manifestations of pharyngeal and laryngeal trouble. He was at a loss to know the cause of the trouble, and sent him to New York to see Dr. Austin Flint, Sr., who said he had an aortic aneurism. His health had been tolerable until a week ago, when he suddenly became paralyzed. There is at present no pulsation in carotid artery on left side, but great tenderness in its middle portion; in the right carotid the pulsations are feeble.

Dr. J. G. Westmoreland inquired if the pulsations ceased suddenly.

Dr. Logan did not know as to that.

Dr. Baird: Can the patient write?

Dr. Logan had not tested him, but as the paralysis was of the right side, and the man right-handed, did not suppose he could.

Dr. Baird said it was rare to have an embolus so low down. It was the cerebral arteries which are generally plugged, and often from rheumatic affection of the valves of the heart; the fibrous depositions upon the valves being brushed off by the force of the circulation, they not being obstructed until they reached arteries of a smaller calibre than the carotid. He called attention to the difference between emboli and thrombi, stating that emboli formed in the left cavities of the heart, or in the arteries, producing obstruction in the arterial tree of which the aorta is the trunk. Whereas, thrombi produce obstruction in the vessel where they originate. He asked Dr. Logan if the man could write. His object in doing so was to ascertain the character of the aphasia. One kind is distinguished by loss of speech, depending on defective memory of words; another, when the loss of speech is due to a lesion of a supposed cerebral apparatus of co-ordination for the movements of articulation—the isle of Reid. These two kinds are chiefly distinguished by the circumstance that in the former the patient has lost the ability to write as well as to speak, whereas in the latter he retains the power to write, but cannot articulate. Thought it important to exercise or stimulate the muscles after all acute brain trouble had passed. He knew of no remedy so potent for this purpose as electricity.

Dr. Logan said he was waiting for nature to establish the collateral circulation. Was giving medicine expectantly, and did not hope for any permanent improvement. He takes fluid aliment entirely, and swallows with tolerable ease.

Dr. Ford: The absence of pulsation in the left carotid can be very easily accounted for, when we consider the tumor as being situated at the origin of that artery. The presence of the aneurism on that vessel would interfere with its free circulation. Being located beyond the origin of the right carotid, the circulation of that vessel is not interrupted in the least.

Dr. Baird said that another difference between thrombosis and embolism was that paralysis from thrombi came on gradually, and not suddenly.

Dr. Logan said there was no circulation in right radial artery, but that in the left it was about natural.

Academy adjourned.

